# Biosynthesis of *L*‐5‐methyltetrahydrofolate by genetically engineered *Escherichia coli*

**DOI:** 10.1111/1751-7915.14139

**Published:** 2022-09-07

**Authors:** Yubo Wang, Meng Zhang, Lexin Li, Jihong Yi, Jiyu Liang, Shuning Wang, Ping Xu

**Affiliations:** ^1^ State Key Laboratory of Microbial Technology Microbial Technology Institute, Shandong University Qingdao China; ^2^ State Key Laboratory of Microbial Metabolism, School of Life Sciences and Biotechnology Shanghai Jiao Tong University Shanghai China

## Abstract

*L*‐5‐Methyltetrahydrofolate (*L*‐5‐MTHF) is the only biologically active form of folate in the human body. Production of *L*‐5‐MTHF by using microbes is an emerging consideration for green synthesis. However, microbes naturally produce only a small amount of *L*‐5‐MTHF. Here, *Escherichia coli* BL21(DE3) was engineered to increase the production of *L*‐5‐MTHF by overexpressing the intrinsic genes of dihydrofolate reductase and methylenetetrahydrofolate (methylene‐THF) reductase, introducing the genes encoding formate‐THF ligase, formyl‐THF cyclohydrolase and methylene‐THF dehydrogenase from the one‐carbon metabolic pathway of *Methylobacterium extorquens* or *Clostridium autoethanogenum* and disrupting the gene of methionine synthase involved in the consumption and synthesis inhibition of the target product. Thus, upon its native pathway, an additional pathway for *L*‐5‐MTHF synthesis was developed in *E. coli*, which was further analysed and confirmed by qRT‐PCR, enzyme assays and metabolite determination. After optimizing the conditions of induction time, temperature, cell density and concentration of IPTG and supplementing exogenous substances (folic acid, sodium formate and glucose) to the culture, the highest yield of 527.84 μg g^−1^ of dry cell weight for *L*‐5‐MTHF was obtained, which was about 11.8 folds of that of the original strain. This study paves the way for further metabolic engineering to improve the biosynthesis of *L*‐5‐MTHF in *E. coli*.

## Graphical abstract

*L* ‐5‐Methyltetrahydrofolate (*L*‐5‐MTHF) is the only biologically active form of folate in the human body. Production of L‐5‐MTHF by using microbes is an emerging consideration for green synthesis. Here, *Escherichia coli* BL21(DE3) was engineered to increase the production of L‐5‐MTHF. After optimizing the conditions and supplementing exogenous substances, the highest yield of 527.84 µg∙g^‐1^ of dry cell weight for L‐5‐MTHF was obtained, which was about 11.8 folds of that of the original strain.

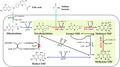

## INTRODUCTION

*L*‐5‐methyltetrahydrofolate (*L*‐5‐MTHF) is the main form of folate in serum and is the only biologically active form of folate in the human body. It is also the only folate molecule that can penetrate the blood–brain barrier (Wu & Pardridge, 1999). *L*‐5‐MTHF is necessary for various methylation events, including the conversion of homocysteine to methionine, the biosynthesis of glycine from serine and the biosynthesis of DNA precursor molecules (Ebara, 2017; Ferrazzi et al., 2020). Folate deficiency is associated with several diseases, such as neural tube defects, megaloblastic anaemia, cardiovascular diseases and cancers (Ferrazzi et al., 2020; Green & Datta, 2017; Jones et al., 2012; Scaglione & Panzavolta, 2014; Vidmar et al., 2020). *L*‐5‐MTHF can be directly absorbed and utilized, thus preventing the potential negative effects of unconverted folic acid in the peripheral circulation (Scaglione & Panzavolta, 2014). Mutation of the methylenetetrahydrofolate reductase (MTHFR) gene in humans decreases the activity of the MTHFR, which causes a reduction in the content of *L*‐5‐MTHF in the body and increases the concentration of plasma homocysteine, leading to hyperhomocysteinemia (Boyi et al., 2013). Therefore, *L*‐5‐MTHF must be taken directly to prevent folate deficiency in this group of people (Vidmar et al., 2020).

Currently, the synthesis of 5‐MTHF is performed using mainly a chemical method that includes the steps of reducing folic acid to tetrahydrofolate (THF) through catalytic hydrogenation or borohydride reduction, the methylation of THF to methylene‐THF using formaldehyde and the reduction of methylene‐THF to methyl‐THF using borohydride (Huennekens et al., 1963; Kitts & Liu, 2015; Scrimgeour & Vitols, 1966; Zakrzewski & Sansone, 1971). However, the 5‐MTHF produced in this way is isomeric, and chiral resolution is needed to obtain the biologically active *L*‐5‐MTHF form (Choi & Schilsky, 1988). This procedure is complicated and requires toxic chemicals and strong reductants, which may cause production safety problems and environmental pollution. Microorganisms can naturally synthesize the bioactive *l*‐5‐MTHF (Lu et al., 2019; Shane & Stokstad, 1977). The production of *l*‐5‐MTHF using microorganisms has recently been considered as an environmentally friendly and sustainable method although the yield is not high. Several microorganisms have been engineered to improve the production of *L*‐5‐MTHF. Increased production of *L*‐5‐MTHF in *Bacillus subtilis* has been achieved by facilitating the synthesis of direct precursors of the dihydrofolate and repressing related competitive and catabolic pathways (Yang et al., 2020). The same group recently further improved the yield of *L*‐5‐MTHF through modular engineering and global regulation of gene expression involved in the synthesis of *L*‐5‐MTHF (Yang et al., 2022). As a widely used lactic acid bacteria, *Lactococcus lactis* is favourable for producing *L*‐5‐MTHF. By strengthening the supply of folate and NADPH via overexpression of some key enzymes such as glucose‐6‐phosphate dehydrogenase, the production of *L*‐5‐MTHF in *L. lactis* was successfully improved (Lu et al., 2019). *Escherichia coli* has also been receiving much attention because of its clear genetic background, efficient genetic operation, simple culture conditions and economic large‐scale fermentation. A few studies on the production of *L*‐5‐MTHF using *E. coli* focussed mainly on the overexpression of the MTHFR gene (*metF*) or glycine decarboxylase gene (*gcvP*) to increase the production of *L*‐5‐MTHF (Han et al., 2013; Liu et al., 2011, 2012; Shao et al., 2013). Studies on the production of *L*‐5‐MTHF by engineered microorganisms are summarized in Table 1, and these studies have demonstrated the promise of development in the production of *L*‐5‐MTHF using engineered microorganisms. However, the yields remain low, especially in *E. coli*.

**TABLE 1:** Studies on the production of *L*‐5‐MTHF by engineered microorganisms^a^

Strains	Key strategies	Medium components	Culture conditions	Yield	References
*B. subtilis*	Replacing *yitJ* with *E. coli metF*, disrupting *purU*, overexpressing *dfrA*, *folC*, *pabB*, *folE* and *yciA*, and repressing the transcription of *pheA* by CRISPRi	10 g L^−1^ yeast extract, 20 g L^−1^ tryptone, 20 g L^−1^ NaCl, 60 g L^−1^ glucose, 1 g L^−1^ glutamate and 0.3 g L^−1^ *p*ABA	37°C, 18 h	1.78 mg L^−1^	Yang et al. (2020)
*B. subtilis*	Strengthening the supply of GTP and *p*ABA by modular engineering, and globally regulating the expression of the key genes for 5‐MTHF synthesis	12 g L^−1^ yeast extract, 6 g L^−1^ tryptone, 6 g L^−1^ (NH_4_)_2_SO_4_, 12.5 g L^−1^ K_2_HPO_4_·3H_2_O, 2.5 g L^−1^ KH_2_PO_4_, 60 g/L glucose, 3 g L^−1^ MgSO_4_·7H_2_O, 1.5 g L^−1^ sodium glutamate and 0.3 g L^−1^ *p*ABA	37°C, 16 h	3.41 mg L^−1^	Yang et al. (2022)
*L. lactis*	Overexpressing *metF*, *drfA*, *folE*, *zwf* and *fau*	GM17 medium^a^ supplemented with key precursors of folate, including 50 mg L^−1^ *p*ABA, 300 mg L^−1^ glutamate and 30 mg L^−1^ GTP	30°C^b^	300 μg L^−1^	Lu et al. (2019)
*E. coli* BL21(DE3)	Overexpressing *metF*	LB medium (5.0 g L^−1^ yeast extract, 1.0 g L^−1^ peptone and 10 g L^−1^ NaCl)	28°C, induced by lactose for 6 h	66 μg g^−1^ WCW	Liu et al. (2011)
*E. coli* BL21(DE3)	Overexpressing *metF*	LB medium	37°C, induced by IPTG for 4 h	129 μg g^−1^ WCW^c^	Liu et al. (2012)
*E. coli* BL21(DE3)	Overexpressing *gcvP*	LB medium	37°C, induced by lactose for 6 h	13.383 μg L^−1^	Han et al. (2013)
*E. coli* BL21(DE3) BLPM08	Optimizing the fermentation process through single‐factor and orthogonal experiments	30 g L^−1^ sucrose, 10 g L^−1^ yeast extract, 12.6 g L^−1^ NaCl, 4.2 g L^−1^ K_2_HPO_4_, 0.2 g L^−1^ MgSO_4_ and 10 g L^−1^ NH_4_H_2_PO_4_	37°C, induced by lactose for 6 h	81. 2 μg L^−1^	Shao et al. (2013)
*E. coli* BL21(DE3)	Overexpressing *metF* and *folA* from *E. coli* and *mtdA*, *fch* and *ftfL from M. extorquens* AM1/Overexpressing *metF* and *folA* from *E. coli* and *folD*, *fchA* and *fhs* from *C. autoethanogenum*, and disrupting *metH* from *E. coli*	24 g L^−1^ yeast extract, 12 g L^−1^ peptone, 4 ml L^−1^ glycerin, 12.5 g L^−1^ KH_2_PO_4_, 2.3 g L^−1^ K_2_HPO_4_ and 1.3 g L^−1^ sodium formate/the same medium supplemented with 0.13 g L^−1^ folic acid	25°C, induced by IPTG for 12 h/16 h	527.84 μg g^−1^ DCW (1.24 mg L^−1^)/445.94 μg g^−1^ DCW (0.88 mg L^−1^)	This study

The abbreviations used in the table: DCW, dry cell weight; *drfA*, dihydrofolate reductase; *fau*, 5‐formyltetrahydrofolate cyclo‐ligase; *fch*, formyl‐THF cyclohydrolase; *fchA*, formyl‐THF cyclohydrolase; *fhs*, formate‐THF ligase; *folA*, dihydrofolate reductase; *folC*, folyl‐polyglutamate synthetase; *folD*, methylene‐THF dehydrogenase; *folE*, GTP cyclohydrolase I; *ftfL*, formate‐THF ligase; *gcvP*, geneglycine decarboxyl; *metF*, methylenetetrahydrofolate reductase; *metH*, methionine synthase; *mtdA*, methylene‐THF dehydrogenase; *p*ABA, *p*‐aminobenzoic acid; *pab*B, para‐aminobenzoate synthase; *pheA*, prephenate dehydratase; *purU*, formyltetrahydrofolate decarboxylase; WCW, wet cell weight; *yciA*, zinc‐independent GTP cyclohydrolase IB; *yitJ*, bifunctional 5,10‐methylenetetrahydrofolate reductase/homocysteine *S*‐methyltransferase; *zwf*, glucose‐6‐phosphate dehydrogenase.

^a^ GM17 medium: 5.0 g L^−1^ pancreatic digest of casein, 5.0 g L^−1^ enzymatic digest of soybean meal, 5.0 g L^−1^ beef extract, 2.5 g L^−1^ yeast extract, 0.5 g L^−1^ L‐ascorbic acid, 0.25 g L^−1^ magnesium sulphate, 19.0 g L^−1^ sodium glycerophosphate, 5.0 g L^−1^ lactose and 5.0 g L^−1^ glucose.

^b^ No fermentation time reported.

^c^ The yield was not reported, which is extrapolated from the publications of the same group (Liu et al., 2011, 2012).

**FIGURE 1:**
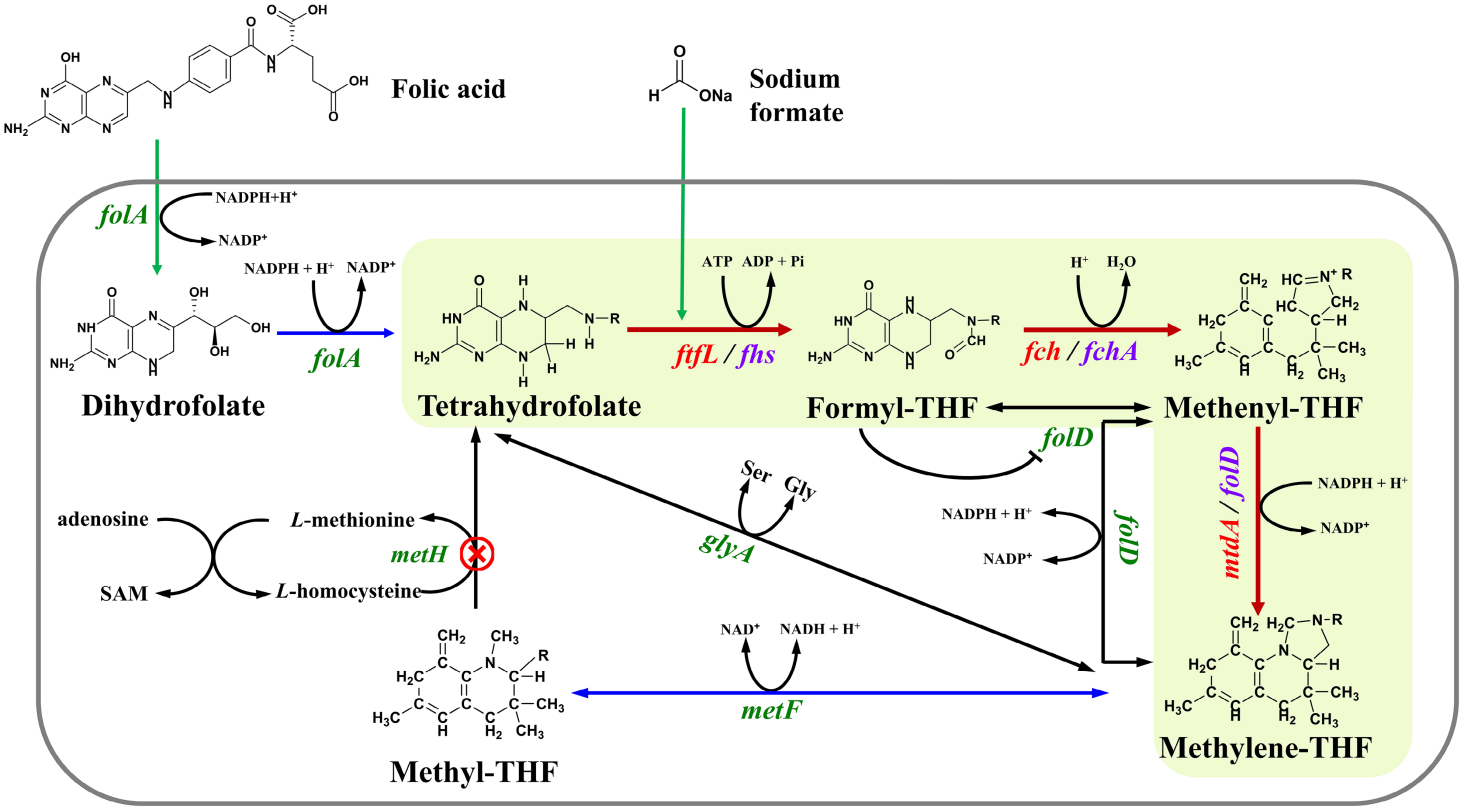
Construction of an additional pathway for the synthesis of *L*‐5‐MTHF in *E. coli*. Red arrows indicate heterologous pathways, black arrows indicate native pathway of *E. coli*, green arrows indicate the substance that needs to be added to the medium, blue arrows indicate the overexpression of the endogenous gene of *E. coli* BL21(DE3), the red 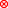 indicates genes knocked out, and the shape ‘┤’ indicates transcriptional repression of the relevant genes. The red/purple words indicate the enzyme introduced by expressing the exogenous genes from *Methylobacterium extorquens* AM1/*Clostridium autoethanogenum*, the green words represent the endogenous genes of *E. coli* BL21(DE3). *folA*: dihydrofolate reductase (DHFR) encoding gene; *ftfL*/*fhs*: formate‐THF ligase (FTHFL) encoding gene; *fch*/*fchA*: formyl‐THF cyclohydrolase (FTHFC) encoding gene; *mtdA*/*folD*: methylene‐THF dehydrogenase (MTHFD) encoding gene; *metF*: methylenetetrahydrofolate reductase (MTHFR) encoding gene; *metH*: methionine synthase (MTRR) encoding gene; *glyA*: serine hydroxymethyltransferase (SHMT) encoding gene; SAM: *S*‐adenosylmethionine. The introduced steps are shaded in lime.

In this study, we tried to improve the production of *L*‐5‐MTHF in *E. coli* through pathway engineering. By overexpressing the intrinsic enzymes dihydrofolate reductase (DHFR) and methylene‐THF dehydrogenase (MTHFR) and introducing the genes encoding the enzymes formate‐THF ligase (FTHFL), formyl‐THF cyclohydrolase (FTHFC) and MTHFD from the one‐carbon metabolic pathway of *Methylobacterium extorquens* AM1 or *Clostridium autoethanogenum*, an additional pathway was constructed upon its native pathway to produce *L*‐5‐MTHF (Figure 1). Further optimization of the synthetic conditions and supplementation with folic acid, sodium formate and glucose were also used to increase the yield of *L*‐5‐MTHF. This provides a new approach for improving the production of *L*‐5‐MTHF.

## EXPERIMENTAL PROCEDURES

### Bacterial strains, plasmids and culture conditions

*Escherichia coli* BL21(DE3), which was used as the original strain, was cultured in an LB medium at 37°C, and the medium was composed of yeast extract 5.0 g L^−1^, peptone 1.0 g L^−1^, NaCl 10 g L^−1^. The TB medium (yeast extract 24.0 g L^−1^, peptone 12.0 g L^−1^, glycerin 4 ml L^−1^, KH_2_PO_4_ 12.5 g L^−1^, K_2_HPO_4_ 2.3 g L^−1^) is used for *L*‐5‐MTHF synthesis of the engineered strains. *Clostridium autoethanogenum* DSM 10061 was purchased from Deutsche Sammlung von Mikroorganismen und Zellkulturen GmbH (DSMZ) and cultivated under strict anaerobic conditions, as described previously (Wang et al., 2013). *Methylobacterium extorquens* AM1 (ATCC 14718) was obtained from the American Type Culture Collection (ATCC) and was grown in a minimal medium, as described previously (Peyraud et al., 2009). The pETDuet‐1 and pACYCDuet‐1 plasmids were used to express various genes, whereas the pTKRed, pKD4 and pCP20 plasmids were used for gene disruption. All strains and plasmids used are listed in Table 4. When required, antibiotics [chloramphenicol (25 mg L^−1^), ampicillin (100 mg L^−1^), spectinomycin (50 mg L^−1^) and kanamycin (50 or 25 mg L^−1^)] were added to the medium.

### DNA manipulation

All operations were carried out according to the kit protocols. *TransStart*® *FastPfu* DNA Polymerase (Transgen Biotech) was used for PCR amplification, the TIANprep Mini Plasmid Kit (Tiangen Biotech) was used for plasmid extraction, and the TIANamp Bacteria DNA Kit (Tiangen Biotech) was used for the isolation of genomic DNA.

### Construction of recombinant plasmids

The *metF* gene (encoding MTHFR; the locus tag from GenBank, b3941, will be used henceforth) and *folA* gene (encoding DHFR; b0048) from *E. coli* were amplified using the genomic DNA of *E. coli* as the template. Three genes, i.e. *mtdA* (encoding methylene‐THF dehydrogenase, MTHFD; MexAM1_META1p1728), *fch* (encoding formyl‐THF cyclohydrolase, FTHFC; MexAM1_META1p1729) and *ftfL* (encoding formate‐THF ligase, FTHFL; MexAM1_META1p0329), from *M. extorquens* AM1 were amplified using the genomic DNA of *M. extorquens* AM1 as the template. Moreover, three genes, i.e. *fhs* (encoding FTHFL; CAETHG_RS07850), *fchA* (encoding FTHFC; CAETHG_RS07845) and *folD* (encoding MTHFD; CAETHG_RS07840), from *C. autoethanogenum* were amplified using the genomic DNA of *C. autoethanogenum* as the template. The primers used are listed in Table S1.

The *metF* and *folA* genes were cloned into the MCS1 and MCS2 of the pACYCDuet‐1 plasmid, respectively, to obtain the recombinant plasmid pACYCDuet‐*metF*‐*folA*. The *ftfL* gene was cloned into the MCS1 of the pETDuet‐1 plasmid and the *mtdA* and *fch* genes were cloned together into the MCS2 of the pETDuet‐1 plasmid, to obtain the recombinant plasmid pETDuet‐C1T. The *fhs*, *fchA* and *folD* genes were cloned together into the MCS1 of the pETDuet‐1 plasmid, to construct the recombinant plasmid pETDuet‐WL.

### Disruption of the *metH* gene of *E. coli* BL21(DE3)

Phage λ Red recombination was used to disrupt the *metH* gene in *E. coli* (Datsenko & Wanner, 2000; Song & Lee, 2013). The FRT‐flanked kanamycin resistance gene (*kan)* with an upstream fragment (400 bp) and a downstream fragment (400 bp) of the *metH* gene was prepared via overlapping extension PCR (Bryksin & Matsumura, 2013). The primers used here are listed in Table S1. The upstream P1 fragment and the downstream P2 fragment of the *metH* gene were amplified using primers *metH*_PUF/*metH*_PUR and *metH*_PDF/*metH*_PDR, respectively, and the genomic DNA of *E. coli* as the template. The FRT‐flanked *kan* P3 fragment was amplified using primers *metH*_PKF and *metH*_PKR and the plasmid pKD4 as the template. Using overlapping PCR, the P1, P2 and P3 fragments were connected to obtain the target fragment (Hobert, 2002). The target fragment (10 μl) was mixed with 100 μl of electrocompetent *E. coli* BL21(DE3) cells carrying pTKRed, and the mixed solution was transferred to an ice‐cold 0.2 cm cuvette (Bio‐Rad Laboratories). Electroporation was conducted using a Gene Pulser Xcell electroporation system (Bio‐Rad Laboratories) at 2.5 kV, 25 mF and 200 Ω, immediately followed by the addition of 1 ml of SOC medium (yeast extract 5.0 g L^−1^, peptone 20.0 g L^−1^, NaCl 0.5 g L^−1^, 2.5 mM KCl, 10 mM MgCl, 10 mM MgSO_4_ and 20 mM glucose). The culture was then incubated for 2 h at 37°C. A 100 μl aliquot of the cell suspension was spread onto LB agar containing kanamycin and incubated for 16–18 h at 37°C, to select kanamycin‐resistant recombinants. To eliminate the pCP20 antibiotic resistance gene, a temperature‐sensitive plasmid carrying thermally inducible FLP (flip‐out) recombinase, was finally electroporated into the *kan*‐replacement mutants, and the transformants were selected at 30°C on LB agar containing both ampicillin and chloramphenicol. Subsequently, a few colonies were purified once at 43°C on nonselective LB agar and were then tested for loss of all antibiotic resistance. The loss of the *kan* gene on the chromosome was further confirmed by gene sequencing analysis using the *metH*_PUF and *metH*_PDR primers (Table S1).

### Construction of engineered strains

*E. coli* transformation was performed by chemical transformation. The pACYCDuet‐*metF*‐*folA* plasmid was transformed into *E. coli* BL21(DE3) and *E. coli* BL21(*ΔmetH*) competent cells, to obtain the engineered strains BL21‐*metF*‐*folA* and BL21(*ΔmetH*)‐*metF*‐*folA*, respectively. Plasmids pACYCDuet‐*metF*‐*folA* and pETDuet‐C1T were co‐transformed into *E. coli* BL21(DE3) and *E. coli* BL21(*ΔmetH*) competent cells, to obtain the engineered strains BL21‐C1T and BL21(*ΔmetH*)‐C1T, respectively. Plasmids pACYCDuet‐*metF*‐*folA* and pETDuet‐WL were co‐transformed into *E. coli* BL21(DE3) competent cells, to obtain the BL21‐WL strain.

### Growth of engineered strains and extraction of *L*‐5‐MTHF

A basic growth test of the engineered strains was performed using a microplate reader (BioTek Instruments) by inoculating the overnight activated culture (1% inoculum) in 96‐well plates containing 500 μl of TB medium at 37°C. Antibiotics were added as required, and the optical density was measured at 600 nm every hour. For other tests, they were cultivated using shaking flasks. Routinely, the engineered strains were inoculated into 100 ml of TB medium that was supplemented with 0.013 g of folic acid and 0.13 g of sodium formate, then cultivated at 37°C with a rotation speed of 220 rpm. When the OD_600nm_ value reached 0.8, IPTG was added to a final concentration of 0.8 mM. Subsequently, the temperature was lowered to 30°C and the rotation speed was lowered to 110 rpm. After induction for 12 h, the cells were harvested by centrifugation at 4°C and 3400 *g* for 10 min. Because *L*‐5‐MTHF is located intracellularly, it is necessary to break cells to extract the product. The harvested cells were resuspended in 4 ml of extraction buffer (50 mM Tris–HCl, pH 7.2, oxygen‐free) containing 1.0% ascorbic acid and 0.1% β‐mercaptoethanol in an anaerobic glove box and sealed in serum bottles with butyl rubber, to prevent the oxidation of *L*‐5‐MTHF (Yang et al., 2020). The cell suspension was incubated in boiling water for 10 min and immediately cooled in an ice bath. Cell debris was removed by centrifugation at 4°C and 20,000 *g* for 15 min. One millilitre of the supernatant was transferred into a 1.5 ml centrifuge tube, and then, 50 μl of fresh mouse serum was added to the sample, as a source of *γ*‐glutamyl hydrolase. The mixture was incubated at 37°C for 3 h, to deconjugate the glutamate tail, followed by incubation at 100°C for 5 min, to inactivate the serum and precipitate the protein. Finally, the precipitate was removed by centrifugation at 4°C and 20,000 × *g* for 15 min, followed by further filtration using a 0.2 μm sterile filter.

### Determination and identification of *L*‐5‐MTHF by HPLC and LC–MS

High‐performance liquid chromatography (HPLC) analysis of *L*‐5‐MTHF was performed using an LC‐20AT system (Shimadzu) according to a previous description, with modifications (Jastrebova et al., 2003). Briefly, a mobile phase containing 93% 33 mM potassium phosphate (pH 3.0) and 7% acetonitrile was pumped through a ChromCore C18 column (250 × 4.6 mm; particle size, 5 μm; NanoChrom) at a flow rate of 0.5 ml·min^−1^, and a fluorescence detector (290/356 nm) was used. The column temperature was set to 30°C, and the injection volume was 20 μl. A standard curve was prepared to quantify the content of the product in the samples. High‐performance liquid chromatography–tandem mass spectrometry (HPLC‐MS/MS) was performed on a rapid‐separation liquid chromatography system [UltiMate3000 Ultra‐HPLC (UHPLC); Dionex] coupled with an electrospray ionization‐quadrupole time of flight (ESI‐Q‐TOF) mass spectrometer (Impact HD; Bruker Daltonics) using a UV detector (290 nm). Chromatographic separations were performed on a ChromCore C18 column (250 × 4.6 mm; particle size, 5 μm; NanoChrom) at 30°C with a mobile phase system containing 8 mM formic acid in Milli‐Q filtered water and acetonitrile. The remaining detection conditions were the same as those described for the HPLC analysis.

### Transcriptional analysis of genes

The expression levels of exogenous genes in the engineered *E. coli* strains, including *mtdA*, *fch* and *ftfL* from *M. extorquens* AM1 and *fhs*, *fchA* and *folD* from *C. autoethanogenum*, were determined using quantitative real‐time reverse transcription‐PCR (qRT‐PCR). The primers used in the qRT‐PCR analysis are listed in Table S1. The culture conditions of the engineered strains were the same as those described above. The engineered strains were cultured in a TB medium at 37°C to an OD_600nm_ value of 0.8, and 1 ml of culture was taken as the sample before induction. Subsequently, IPTG at a final concentration of 0.6 mM was added for induction for 12 h at 25°C, and 1 ml of culture was taken and diluted with sterile saline to an OD_600nm_ value of 0.8, then used as the sample after the induction. The harvested cells were used for RNA extraction with an EasyPure RNA kit (Transgen Biotech). The cDNA was synthesized using 5× All‐In‐One MasterMix with the AccuRT genomic DNA removal kit (ABM) and was used as the template for qPCR analysis. qPCR was carried out in a LightCycler 480 instrument with the TransStart Top Green qPCR supermix (Transgen Biotech). The *16S* rRNA gene was used as an internal reference. Three replicates for each sample were used for qPCR, after which the average threshold cycle (*C*_*T*_) was calculated for each sample. Using the *C*_*T*_ values of the target genes before induction as the baseline, the relative fold changes in gene expression were calculated using the 2^−Δ*C*T^ method (Wang et al., 2019).

### Enzymatic assay

For the determination of the MTHFD‐specific activity, the reaction was carried out in a 1.5 ml quartz anaerobic cuvette sealed with a rubber stopper and the gas phase was 100% N_2_. The assay mixtures contained 100 mM MOPS‐KOH (containing 2 mM DTT, pH 6.5), 40 mM NADP^+^, 200 mM formaldehyde and 10 mM THF. After starting the reaction using the crude enzyme, the formation of NADPH and methylene‐THF was monitored at 350 nm using an *ε* of 30.5 mM^−1^ cm^−1^ (NADPH *ε* = 5.6 mM^−1^ cm^−1^, methylene‐THF *ε* = 24.9 mM^−1^ cm^−1^) (Mock et al., 2015). For the determination of the MTHFR‐specific activity, the assay mixture contained 50 mM Tris–HCl (with 2 mM DTT, pH 7.4), 10 mM tetrahydrofolate, 200 mM formaldehyde and 0.2 mM NADH. After starting the reaction using the enzyme, the reduction in NAD^+^ was monitored at 350 nm (*ε* = 6.2 mM^−1^ cm^−1^) (Mock et al., 2014; Wang et al., 2013). One unit (U) was defined as the amount of enzyme required to form or consume 1 μmol of product or substrate. Protein concentration was determined using the Bradford method with bovine serum albumin as the standard (Bradford, 1976).

### Preliminary optimization of the conditions for *L*‐5‐MTHF synthesis

The induction time of the engineered strains was first tested for 4, 8, 12, 16 and 20 h. The other cultural conditions were as described above. Under the optimal induction time, the induction temperature of the engineered strains was then tested at 20, 25, 30 and 37°C. Moreover, the effects of different IPTG concentrations (0.4, 0.6, 0.8 and 1.0 mM) on the production of *L*‐5‐MTHF were studied. The effects of supplementation with folic acid, sodium formate and glucose on the production of *L*‐5‐MTHF by the engineered strains were analysed under the optimal induction conditions obtained above. The experiment was divided into five groups. In the first group, no substance was added to the TB medium. The second group was supplemented only with folic acid (0.13 g L^−1^) in the TB medium, the third group was supplemented only with sodium formate (1.3 g L^−1^) in the TB medium, the fourth group was supplemented with both folic acid and sodium formate in the TB medium, and the fifth group was supplemented with folic acid, sodium formate and glucose (5 g L^−1^) in the TB medium. Three shaking flasks for each group were used in parallel, and the production of *L*‐5‐MTHF was determined by HPLC, as described above.

## RESULTS

### Construction and screening of engineered strains for *L*‐5‐MTHF production

To improve the production of *L*‐5‐MTHF in *E. coli*, its intrinsic *metF* gene (encoding MTHFR) and *folA* gene (encoding DHFR) were amplified, cloned into the pACYCDuet‐1 plasmid and further introduced into *E. coli* BL21(DE3) to enhance their expression. In addition, three genes, i.e. *mtdA* (encoding MTHFD), *fch* (encoding FTHFC) and *ftfL* (encoding FTHFL), from *M. extorquens* AM1, which are involved in the C1 transfer pathway; and three genes, i.e. *fhs* (encoding FTHFL), *fchA* (encoding FTHFC) and *folD* (encoding MTHFD), from *C. autoethanogenum*, which are involved in the Wood–Ljungdahl pathway, were chosen as two sets of candidates and cloned into pETDuet‐1, respectively. By introducing these genes into *E. coli* BL21(DE3), an additional *L*‐5‐MTHF synthesis pathway was constructed (Figure 1). In this pathway, folic acid is first reduced to THF by DHFR via two steps of reductive reactions (Blakley & Benkovic, 1984). THF then combines the C1 group, generating methylene‐THF under the actions of FTHFL, FTHFC and MTHFD (Chistoserdova et al., 2003; Drake et al., 2008). Finally, methylene THF is reduced to *L*‐5‐MTHF by MTHFR (Trimmer et al., 2001). Moreover, to block *L*‐5‐MTHF consumption by methionine synthase (MTRR), its encoding gene (*metH*) was disrupted in *E. coli*. After transforming one or two of the recombinant plasmids into *E. coli* BL21(DE3) and BL21(*ΔmetH*), six engineered strains were constructed.

The growth of the six engineered strains was first evaluated by measuring the OD_600nm_ of the cultures using a microplate reader and 96‐well plates. The growth curve showed that the growth trend of the various engineered strains was basically the same as that of the wild‐type strains without adding IPTG, folic acid, sodium formate and glucose (Figure S1), indicating that the introduced exogenous genes and knockout of the *metH* gene had no obvious effect on the growth of the strains.

Subsequently, the formation of the *L*‐5‐MTHF product was detected and confirmed by HPLC and LC–MS. Under the detection conditions used here, the retention time of *L*‐5‐MTHF for cell extracts was found to be 19.37 min (the retention time for 5‐MTHF standard was 19.38 min, Figure S2A), and the target peak was well separated from other impurity peaks (Figure S2B), which was conducive to quantify the production of *L*‐5‐MTHF. Concomitantly, the peaks of THF and methenyl‐THF could also be detected. To further verify the correctness of the target product, the sample from the engineered strain BL21‐WL was detected by LC–MS. A peak with an *m/z* of 460.1953 was detected, which was identical to the theoretical mass of the protonated form [M + H]^+^ of *L*‐5‐MTHF at *m/z* 460.1939, confirming that *L*‐5‐MTHF was present in the cell extracts of the engineered strain (Figure S2C,D).

**FIGURE 2:**
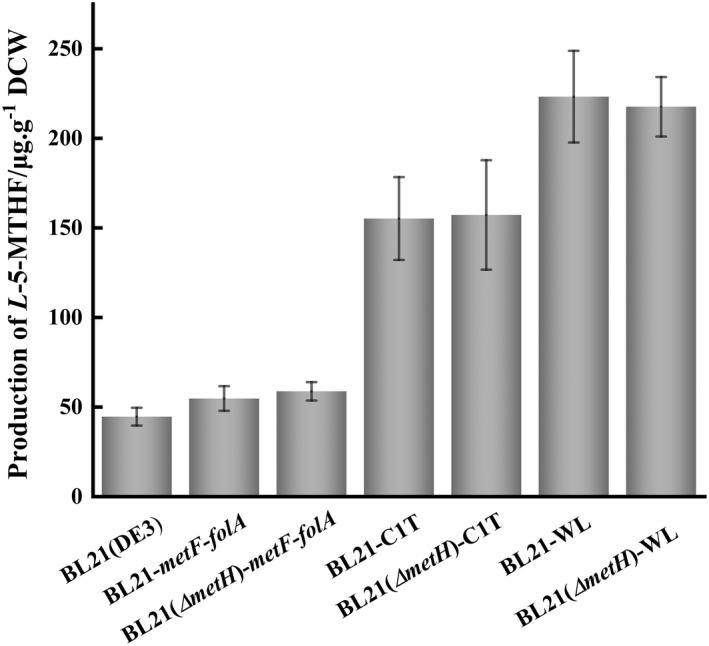
Production of *L*‐5‐MTHF in the engineered strains BL21‐*metF*‐*folA*, BL21(*ΔmetH*)‐*metF*‐*folA*, BL21‐C1T, BL21(*ΔmetH*)‐C1T, BL21‐WL and BL21(*ΔmetH*)‐WL. The six strains were cultured in TB medium for 12 h. The production of *L*‐5‐MTHF was determined with HPLC. All data were the means of triplicate experiments, and the error bars indicate the standard deviations of the data.

Furthermore, the yield of the *L*‐5‐MTHF in the engineered strains was measured using HPLC. The BL21‐*metF*‐*folA* and BL21(*ΔmetH*)‐*metF*‐*folA* engineered strains, which overexpressed the *metF* and *folA* genes, produced 192.60 and 199.98 μg L^−1^ of *L*‐5‐MTHF, respectively, which was slightly lower than the yield of the original strain, *E. coli* BL21(DE3) (233.85 μg L^−1^). When the data were calculated based on the dry cell weight (DCW) biomass, the *L*‐5‐MTHF yield of BL21‐*metF*‐*folA* was 54.79 μg g^−1^ DCW, and the yield of BL21(*ΔmetH*)‐*metF*‐*folA* was 58.78 μg g^−1^, which was 22.79% and 31.73% higher than that of the original strain, *E. coli* BL21(DE3) (44.62 μg g^−1^ DCW) (Figure 2). This difference may result from the inhibition of the growth of the engineered strains by IPTG (Figure S3). When the *metF* and *folA* genes were overexpressed, and the *mtdA*, *fch* and *ftfL* genes were introduced from *M. extorquens* AM1, the yield of the BL21‐C1T and BL21(*ΔmetH*)‐C1T strains reached 155.26 and 157.26 μg g^−1^ of DCW, respectively, which was about 3.5‐fold the yield of the original strain, *E. coli* BL21(DE3) (Figure 2); when converting to the production per litre of culture, the yields were 2.4‐ and 3.0‐fold the yield of the original strain, respectively, reaching 559.38 and 697.54 μg L^−1^. Moreover, the replacement of the *mtdA*, *fch* and *ftfL* genes with the *fhs*, *fchA* and *folD* genes from *C. autoethanogenum* led to a yield of BL21‐WL and BL21(*ΔmetH*)‐WL of 223.29 and 217.65 μg g^−1^ of DCW (Figure 2); when converting to the yield per litre of culture, the yield reached 647.88 and 737.08 μg L^−1^, which was 2.8‐fold and 3.15‐fold the yield of the original strain.

By comparing the yield of *L*‐5‐MTHF in the constructed engineered strains, the two strains with the highest yield of DCW, i.e. BL21‐WL and BL21(*ΔmetH*)‐C1T, were selected for further study, including the analysis of their pathways for the synthesis of *L*‐5‐MTHF and the optimization of the conditions for *L*‐5‐MTHF production.

### Analysis of the synthetic pathway of *L*‐5‐MTHF in engineered strains

**FIGURE 3:**
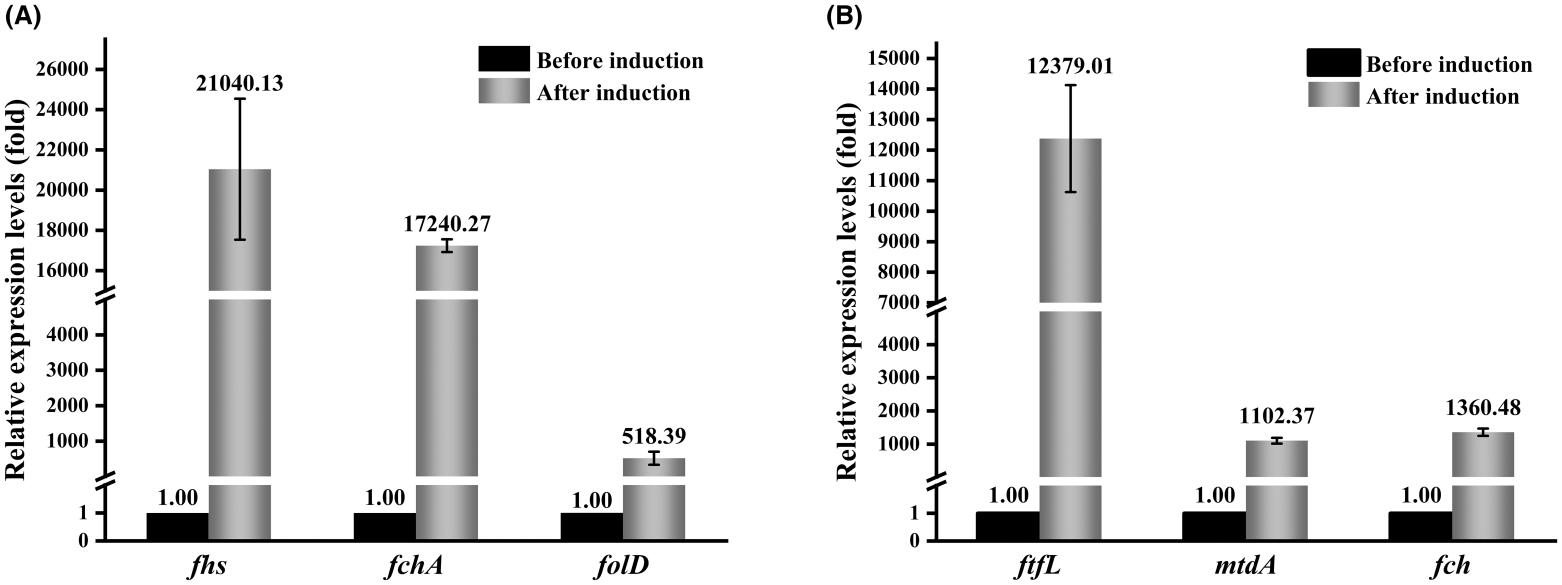
Expression levels of the exogenous genes in BL21‐WL (A) and BL21(*ΔmetH*)‐C1T (B), as assessed by qRT‐PCR analysis (fold‐change). The 16*S* rRNA gene was used as an internal reference, and the *C*_*T*_ values of the exogenous genes before induction in TB medium were used as the baseline. The *fhs*, *fchA* and *folD* genes encode formate‐THF ligase, formyl‐THF cyclohydrolase and methylene‐THF dehydrogenase from the Wood–Ljungdahl pathway of *Clostridium autoethanogenum*, respectively. The *ftfL*, *fch* and *mtdA* genes encode formate‐THF ligase, formyl‐THF cyclohydrolase and methylene‐THF dehydrogenase from the C1 transfer pathway of *Methylobacterium extorquens* AM1, respectively. Values are the mean of three replicates, with standard deviation, as calibrated using the 2^−ΔCt^ method.

The operation of the constructed synthetic pathway for *L*‐5‐MTHF production depended on the expression of the introduced genes in the strains. For the purpose of detecting the expression pattern of the exogenous genes in the BL21‐WL and BL21(*ΔmetH*)‐C1T strains, quantitative real‐time reverse transcription‐PCR (qRT‐PCR) was applied to analyse the transcriptional levels of *mtdA*, *fch* and *ftfL* from *M. extorquens* AM1 and *fhs*, *fchA* and *folD* from *C. autoethanogenum*. As shown in Figure 3, the transcriptional levels of the exogenous genes in strains BL21‐WL and BL21(*ΔmetH*)‐C1T were greatly increased after induction by IPTG, indicating that the exogenous genes successfully overexpressed in the two engineered strains. Nevertheless, the transcriptional levels of the MTHFD‐encoding genes (*folD* and *mtdA*) in the two engineered strains were lower than those of the FTHFL‐encoding genes (*fhs* and *ftfL*) and the FTHFC‐encoding genes (*fchA* and *fch*), with the difference being nearly 40 times in BL21‐WL.

To further analyse the metabolism of the constructed *L*‐5‐MTHF synthesis pathway, the content of the intermediate metabolites in the pathway was also measured. Methenyl‐THF and THF were successfully detected in the engineered strains by HPLC, and their retention times were 14.99 and 16.45 min, respectively (Figure S2B), which was helpful for quantifying them in the cell extracts of the engineered strains. As shown in Table 2, the amount of methenyl‐THF in the engineered strains was much higher than that of THF and *L*‐5‐MTHF, especially in the BL21‐WL and BL21(*ΔmetH*)‐C1T strains. The low accumulation of THF indicates that DHFR, FTHFL and FTHFC should not be the rate‐limiting enzymes in the constructed *L*‐5‐MTHF synthesis pathway. In the pathway, methenyl‐THF is reduced by MTHFD to produce methylene‐THF, which is then further reduced by MTHFR to produce *L*‐5‐MTHF (Figure 1). The large accumulation of methenyl‐THF observed here suggests that MTHFD and MTHFR may be the rate‐limiting enzymes in BL21‐WL and BL21(*ΔmetH*)‐C1T. A subsequent enzyme assay showed that their specific enzyme activities in BL21‐WL and BL21(*ΔmetH*)‐C1T increased compared with the original *E. coli* strain, BL21(DE3), i.e. by 10–15‐fold for MTHFD and only 0.5–0.6‐fold for MTHFR (Table 3). However, their activities remain relatively low, especially that of MTHFD, which may mainly cause a large accumulation of methenyl‐THF.

**TABLE 2:** Determination of intermediate metabolites

Strains	THF/μg g^−1^	Methenyl‐THF/μg g^−1^	Methyl‐THF/μg g^−1^
*E. coli* BL21(DE3)	6.59 ± 1.17	21711.92 ± 3463.85	14.26 ± 3.87
BL21‐WL	78.12 ± 12.17	26295.09 ± 4850.41	182.81 ± 27.56
BL21(*ΔmetH*)‐C1T	97.48 ± 12.33	24277.17 ± 1978.09	258.07 ± 47.31

**TABLE 3:** Determination of the enzymatic activity of MTHFD and MTHFR

Crude enzymes	Specific enzymatic activity (U/mg)	
	MTHFD	MTHFR
*E. coli* BL21(DE3)	0.003 ± 0.0003	0.073 ± 0.003
BL21‐WL	0.047 ± 0.0007	0.114 ± 0.005
BL21(*ΔmetH*)‐C1T	0.033 ± 0.008	0.177 ± 0.083

**TABLE 4:** Strains and plasmids used in this study

Strain or plasmid	Relevant characteristics	Reference or source
Strains		
*E. coli* BL21(DE3)	F^−^, endA, ompT, hsdS (rK^−^ mK^−^), dcm^+^, galλ, araB::T7 RNAP‐tetA	Novagen
*E. coli* BL21(*ΔmetH*)	*E. coli* BL21(DE3) *metH* gene was knocked out	This work
*Clostridium autoethanogenum* DSM 10061	Anaerobic acetogenic bacterium, G^+^	DSMZ
*Methylobacterium extorquens* AM1	Aerobic methylotrophy model strain, G^−^	ATCC
BL21‐*metF*‐*folA*	*E. coli* BL21(DE3) contains plasmid pACYCDuet‐*metF*‐*folA*	This work
BL21(*ΔmetH*)‐*metF*‐*folA*	*E. coli* BL21(ΔmetH) contains plasmid pACYCDuet‐*metF*‐*folA*	This work
BL21‐C1T	*E. coli* BL21(DE3) contains plasmid pACYCDuet‐*metF*‐*folA* and pETDuet‐C1T	This work
BL21(*ΔmetH*)‐C1T	*E. coli* BL21(*ΔmetH*) contains plasmid pACYCDuet‐*metF*‐*folA* and pETDuet‐C1T	This work
BL21‐WL	*E. coli* BL21(DE3) contains plasmid pACYCDuet‐*metF*‐*folA* and pETDuet‐WL	This work
BL21(*ΔmetH*)‐WL	*E. coli* BL21(*ΔmetH*) contains plasmid pACYCDuet‐*metF*‐*folA* and pETDuet‐WL	This work
Plasmids		
pETDuet‐1	Amp^R^; expression vector contains two multiple cloning sites	Novagen
pACYCDuet‐1	Cm^R^; expression vector contains two multiple cloning sites	Novagen
pTKRed	Sp^R^; help plasmid, Psc101ori, β γ exo (red recombinase)	Kuhlman and Cox (2010)
pKD4	Km^R^; R6Kγ ori, FRT‐*kan*‐FRT, *bla*	Datsenko and Wanner (2000)
pCP20	Amp^R^; help plasmid, Psc101ori, FLP, *bla*, *cat*	Cherepanov and Wackernagel (1995)
pACYCDuet‐*metF*‐*folA*	Cm^R^; pACYCDuet‐1 derivative containing *metF* and *folA* genes from *E. coli*	This work
pETDuet‐C1T	Amp^R^; pETDuet‐1 derivative containing *ftfL*, *mtdA* and *fch* genes from *M. extorquens* AM1	This work
pETDuet‐WL	Amp^R^; pETDuet‐1 derivative containing *fhs*, *fchA* and *folD* genes from *C. autoethanogenum*	This work

### Optimization of the conditions for *L*‐5‐MTHF synthesis

**FIGURE 4:**
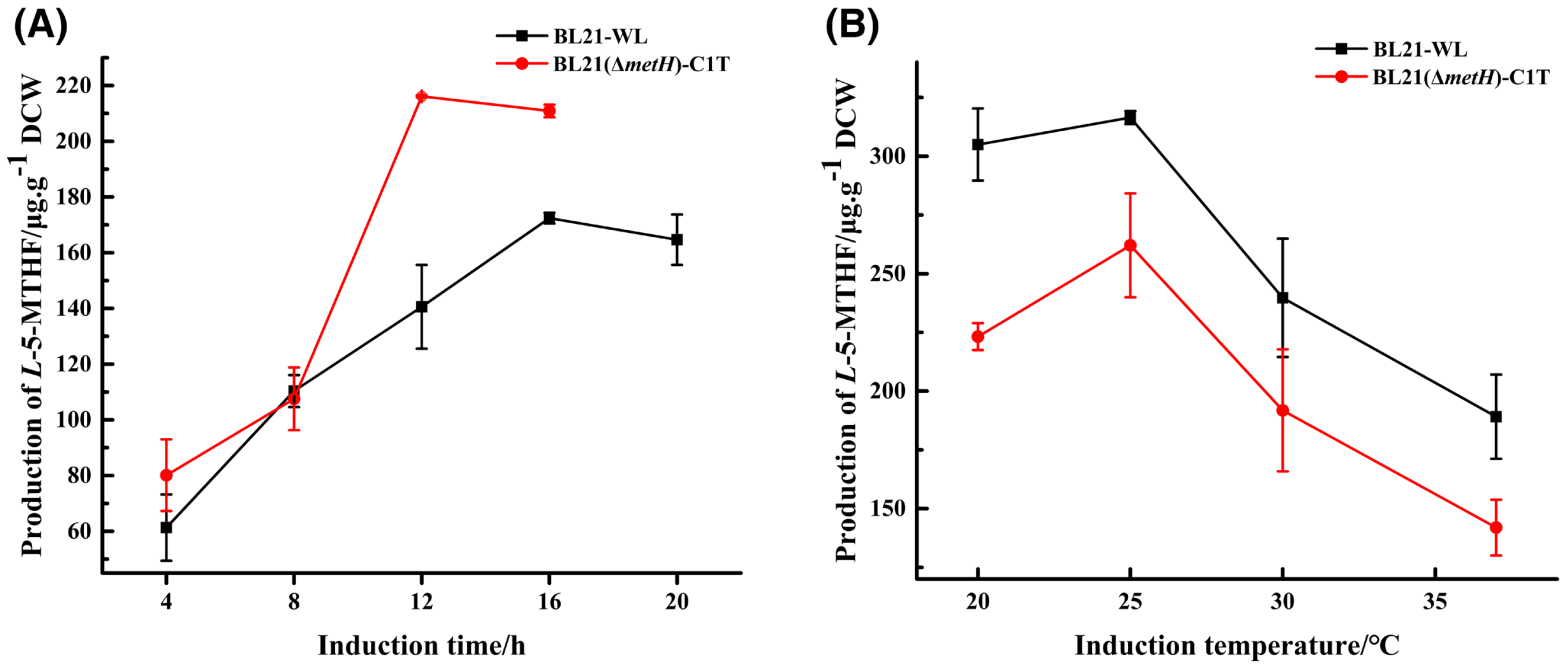
Production of *L*‐5‐MTHF in BL21‐WL and BL21(*ΔmetH*)‐C1T at different induction times and temperatures. (A) depict the production changes in BL21‐WL and BL21(*ΔmetH*)‐C1T at different induction times; (B) depict the production changes in BL21‐WL and BL21(*ΔmetH*)‐C1T at different induction temperatures, respectively. All data were the means of triplicate experiments, and the error bars indicate the standard deviations of the data.

The conditions for the production of *L*‐5‐MTHF by the engineered strains BL21‐WL and BL21(*ΔmetH*)‐C1T were preliminarily optimized. As shown in Figure 4A, the production of *L*‐5‐MTHF by BL21‐WL peaked (172.41 μg g^−1^ of DCW) at an induction time of 16 h, which was 3.9‐fold that of the original strain, *E. coli* BL21(DE3); moreover, the production of *L*‐5‐MTHF in BL21(*ΔmetH*)‐C1T peaked (216.14 μg g^−1^ of DCW) at 12 h, which was 4.8‐fold that of the original strain. The effects of the induction temperature on the production of *L*‐5‐MTHF in BL21‐WL and BL21(*ΔmetH*)‐C1T were also tested. Figure 4B shows that both BL21‐WL and BL21(*ΔmetH*)‐C1T exhibited a higher production of *L*‐5‐MTHF at the induction temperature of 25°C, with a maximum yield of 316.50 μg g^−1^ of DCW and 262.05 μg g^−1^ of DCW, respectively, which was 83.57% and 21.24% higher than that recorded before optimization.

**FIGURE 5:**
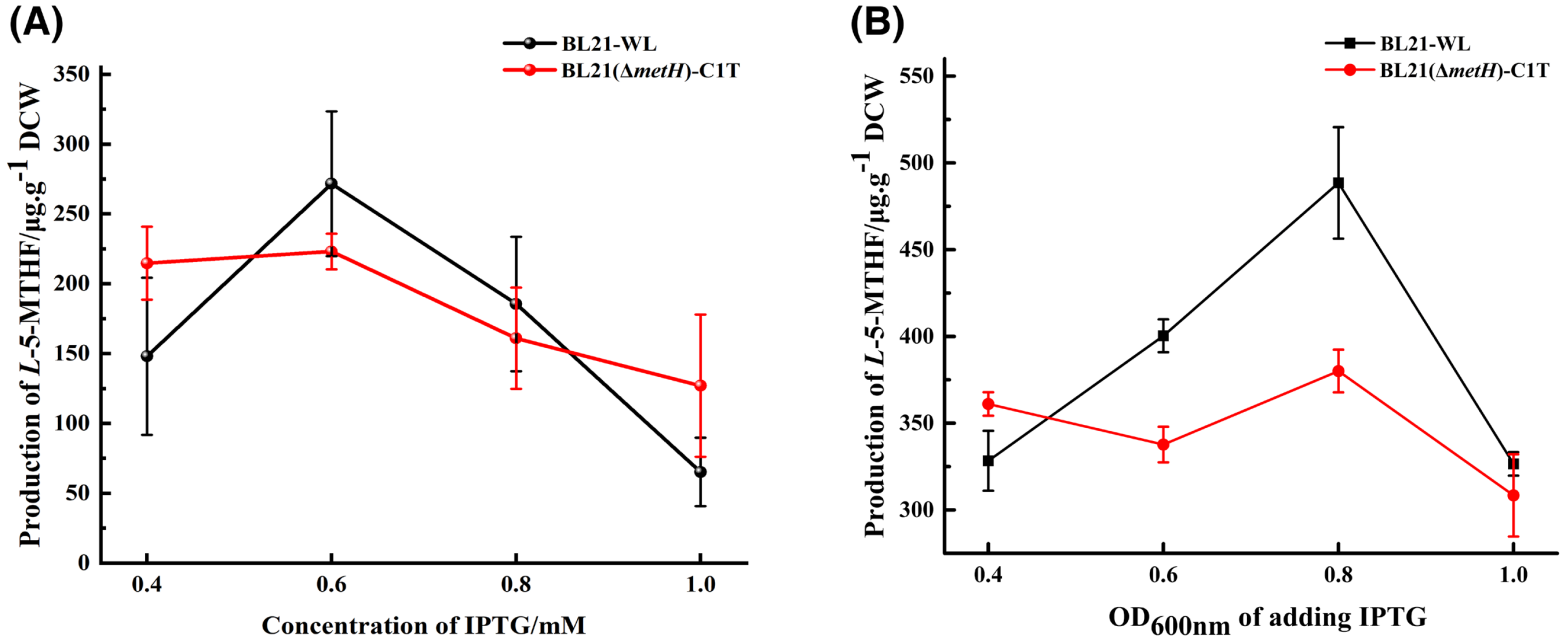
Production of *L*‐5‐MTHF in BL21‐WL and BL21(*ΔmetH*)‐C1T at different final concentrations of IPTG and different OD_600nm_ after adding IPTG. (A) depict the production changes in BL21‐WL and BL21(*ΔmetH*)‐C1T at different final concentrations of IPTG; (B) depict the production changes in BL21‐WL and BL21(*ΔmetH*)‐C1T at different OD_600nm_ values after adding IPTG. All data were the means of triplicate experiments, and the error bars indicate the standard deviations of the data.

Under the optimal induction temperature and time, the effects of IPTG concentration on the production of *L*‐5‐MTHF by the two strains were tested. Figure 5A shows that when 0.6 mM IPTG was added to the culture of BL21‐WL, compared with 0.4 mM IPTG, the production of *L*‐5‐MTHF increased significantly, reaching 271.60 μg g^−1^ of DCW. Continuously increasing the IPTG concentration resulted in a decrease in the yield. In turn, the production of *L*‐5‐MTHF in BL21(*ΔmetH*)‐C1T displayed a similar trend. A final concentration of IPTG of 0.6 mM yielded the highest yield of 223.09 μg g^−1^ of DCW. The production of *L*‐5‐MTHF in BL21‐WL and BL21(*ΔmetH*)‐C1T was further studied after adding IPTG at different growth stages at a final concentration of 0.6 mM. The results of this experiment revealed that adding IPTG at different OD_600nm_ values also had a significant impact on the production of *L*‐5‐MTHF. When the OD_600nm_ value was between 0.4 and 0.8, the yield of BL21‐WL increased with the increase of the OD_600nm_ value of IPTG, with a peak at 469.94 μg g^−1^ of DCW, which was 10.5‐fold that of the original strain, *E. coli* BL21(DE3), representing an increase of 110.46% compared with that recorded before optimization. However, when IPTG was added at an OD_600nm_ value higher than 0.8, the yield decreased significantly (Figure 5B). The results obtained for BL21(*ΔmetH*)‐C1T were similar to those described for BL21‐WL, with a maximum yield of 380.01 μg g^−1^ of DCW obtained when adding IPTG at the OD_600nm_ value of 0.8, which was 8.5‐fold the yield of the original strain and represented an increase of 141.64% compared with that recorded before optimization (Figure 5B). Therefore, the highest yield of *L*‐5‐MTHF can be obtained from BL21‐WL and BL21(*ΔmetH*)‐C1T by adding 0.6 mM IPTG at an OD_600nm_ value of 0.8.

**FIGURE 6:**
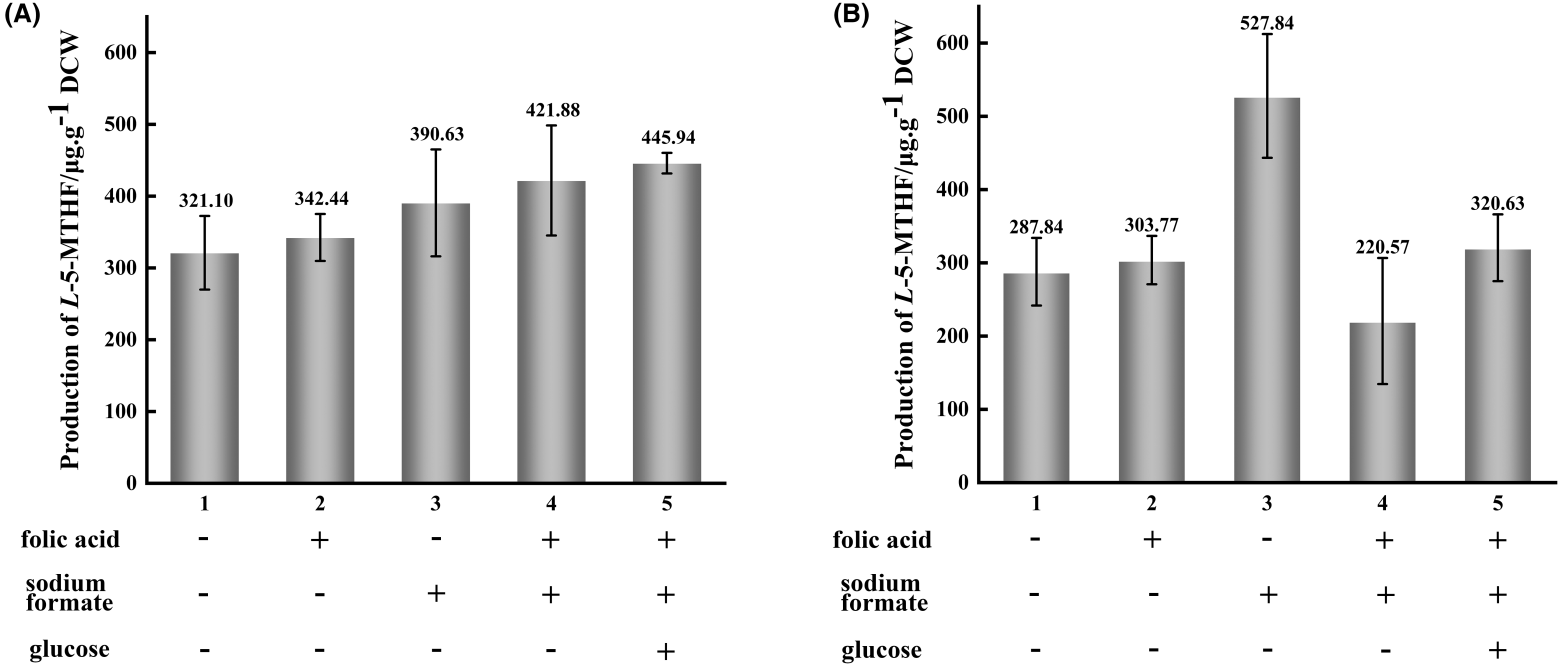
Effect of the addition of various exogenous substances on the production of *L*‐5‐MTHF in BL21‐WL (A) and BL21(*ΔmetH*)‐C1T (B). All data were the means of triplicate experiments.

The FTHFL enzyme from *M. extorquens* AM1 or *C. autoethanogenum* (*E. coli* does not encode this enzyme) catalyses the combination of formate and THF into formyl‐THF, which is an important precursor in the synthesis of *L*‐5‐MTHF. Compared with *L*‐5‐MTHF, folic acid and formate are relatively cheap and easily obtained. Thus, folic acid and sodium formate were added to the culture medium to explore whether the engineered strains were able to assimilate exogenous folic acid and formate to further improve the production of *L*‐5‐MTHF. In addition, the DHFR and MTHFD enzymes in the engineered strains are specific to NADPH (D'Ari & Rabinowitz, 1991; Morrison & Stone, 1988), and intracellular glucose can provide additional NADPH through glucose‐6‐phosphate dehydrogenase (Lu et al., 2019). Therefore, the effects of supplementation with glucose on the synthesis of *L*‐5‐MTHF were also studied. The production of *L*‐5‐MTHF in BL21‐WL and BL21(*ΔmetH*)‐C1T under these conditions was measured in five groups (Figure 6). Other cultural conditions were according to the optimal conditions. Figure 6 shows that folic acid or sodium formate promoted the production of *L*‐5‐MTHF in the engineered strains; when both folic acid and sodium formate were added to the medium, the yield in BL21‐WL was slightly higher than that observed in the presence of only folic acid or sodium formate (Figure 6A), whereas the yield in BL21(*ΔmetH*)‐C1T decreased significantly (Figure 6B). Moreover, the addition of exogenous substances (folic acid, sodium formate and IPTG) had a negative effect on the growth of the engineered strains (Figure S3). The maximum OD_600nm_ value recorded for *E. coli* BL21(DE3) was 6.9, whereas the maximum OD_600nm_ values of BL21‐WL and BL21(*ΔmetH*)‐C1T were 4.7 and 4.6 after the addition of IPTG, respectively. When glucose was also added to the medium, the yield of the two engineered strains was improved compared with that of strains supplemented with both folic acid and sodium formate. However, in BL21(*ΔmetH*)‐C1T, the highest yield was observed in the case of supplementation with sodium formate alone.

In summary, the obtained optimal conditions were as follows: cultivation to an OD_600nm_ value of 0.8 at 37°C with a rotation speed of 220 rpm, followed by induction by adding 0.6 mM IPTG at 25°C with a rotation speed of 110 rpm for 16 h (BL21‐WL) or 12 h [BL21(*ΔmetH*)‐C1T]. It was also found that BL21‐WL yielded the highest production of *L*‐5‐MTHF in the medium supplemented with folic acid, sodium formate and glucose, reaching 445.94 μg g^−1^ DCW (0.88 mg L^−1^), which was 10‐fold that of the original strain. However, it was noted that the production of *L*‐5‐MTHF by BL21‐WL was slightly lower than the highest production of BL21‐WL [469.94 μg g^−1^ DCW (0.97 mg L^−1^)] without the addition of exogenous substances, probably because the addition of folic acid and/or sodium formate negatively affected the cell growth (Figure S3) and/or the expression and activities of some enzymes. The production of *L*‐5‐MTHF by BL21(*ΔmetH*)‐C1T reached its peak [527.84 μg g^−1^ DCW (1.24 mg L^−1^)] in the medium supplemented with sodium formate, which was 11.8‐fold that of the original strain.

## DISCUSSION

To increase the production of *L*‐5‐MTHF, we constructed an additional pathway based on the native pathway used for the synthesis of *L*‐5‐MTHF in *E. coli* (Figure 1). This pathway takes advantage of the related enzymes in strains *M. extorquen*s AM1 or *C. autoethanogenum*, which have a high capacity for the metabolism of one‐carbon compounds (e.g. CO, CO_2_ and methanol) (Chistoserdova et al., 2003; Mock et al., 2015; Ragsdale, 2008). The flavoprotein MTHFR can catalyse irreversibly the reduction of methylene‐THF to methyl‐THF using NADH as the source of reducing equivalents (Trimmer et al., 2001). MTHFR from *E. coli* is a homotetramer composed only of the MetF subunit, whereas MTHFR from *C. autoethanogenum* is an anaerobic enzyme composed of MetF and MetV subunits (Bertsch et al., 2015) that may use reduced 2[4Fe4S]‐ferredoxin as the reducing equivalent (Oppinger et al., 2021; Yi et al., 2021). DHFR from *E. coli* catalyses the reduction of folic acid to dihydrofolate and then reduces dihydrofolate to tetrahydrofolate using NADPH (Iwakura et al., 2006). It is a monomeric protein with two domains, and its properties have been well studied (Morrison & Stone, 1988; Schnell et al., 2004). Consequently, the genes encoding DHFR and MTHFR in *E. coli* were selected for overexpression aimed at enhancing the production of the target product. Considering that the facultative *M. extorquens* AM1 is a model organism that is used for the study of methylotrophic metabolism (Marx, Chistoserdova, & Lidstrom, 2003; Marx, O'Brien, et al., 2003; Peyraud et al., 2009) and has a strong ability to metabolize one‐carbon unit (Marx, Chistoserdova, & Lidstrom, 2003; Marx, O'Brien, et al., 2003), the genes related to the C1 transfer pathway from *M. extorquens* AM1 (*ftfL*, *fch* and *mtdA*) were selected as candidates. *C. autoethanogenum* can ferment CO_2_/H_2_ and CO to acetyl coenzyme A (acetyl‐CoA) via the Wood–Ljungdahl pathway (Drake et al., 2008; Liew et al., 2016; Muller, 2003), further producing acetic acid (Muller, 2003); therefore, the genes involved in the Wood–Ljungdahl pathway from *C. autoethanogenum* (*fhs*, *fchA* and *folD*) were selected. In addition, methionine synthase (MTRR, encoded by *metH*) catalyses the reaction of *L*‐5‐MTHF and homocysteine to produce THF and methionine (Met) (Goulding et al., 1997). MTRR consumes *L*‐5‐MTHF, and the generated Met can repress the activity of MTHFR (Katzen & Buchanan, 1965). Concomitantly, the *S*‐adenosylmethionine (*S*‐AdoMet) generated by Met and adenylate under the action of methyl transferase affords isomorphic inhibition on MTHFR (Matthews, 1986). Therefore, the *metH* gene from *E. coli* BL21(DE3) was knocked out. Based on the above analysis and design, six engineered strains were constructed and used for further research.

When only the native genes (*metF* and *folA*) of *E. coli* were overexpressed, there was a slightly increased yield of *L*‐5‐MTHF (reach 54.79 μg g^−1^ DCW from 44.62 μg g^−1^ DCW). After introducing the exogenous genes from the one‐carbon unit metabolic pathway, the production of *L*‐5‐MTHF by the engineered strains was significantly increased (155.26 μg g^−1^ DCW for BL21‐C1T and 223.29 μg g^−1^ DCW for BL21‐WL), indicating that these genes can promote the synthesis of *L*‐5‐MTHF. However, knockout of the *metH* gene in *E. coli* BL21‐WL did not increase the yield of the *L*‐5‐MTHF [157.26 μg g^−1^ DCW for BL21(*ΔmetH*)‐C1T and 217.65 μg g^−1^ DCW for BL21(*ΔmetH*)‐WL], suggesting that the reaction catalysed by MTRR is not a key factor to affect the production of *L*‐5‐MTHF in the strain.

The increase in the production of *L*‐5‐MTHF was supported by the analysis of the transcriptional levels of the key genes in the constructed pathway (Figure 3). The results of the qRT‐PCR analysis showed that the transcription levels of all the introduced exogenous genes were substantially increased in the engineered strains. *M. extorquens* AM1 has a strong ability to metabolize one‐carbon compounds, and the introduction of its genes (*ftfL*, *fch* and *mtdA*) led to a stronger ability of the engineered strain BL21‐C1T to assimilate sodium formate and to obtain a much higher production of *L*‐5‐MTHF. On the contrary, the addition of folic acid did not obtain a similar result, which may be caused by its inhibitory effect on the pathway. And the addition of glucose was helpful to provide NADPH, which further improved the production of *L*‐5‐MTHF (Figure 6B).

However, the yields of the product *L*‐5‐MTHF in the two engineered strains are still not very high. Some of the exogenous genes introduced into them were tandemly linked following the same promoter of the plasmid (e.g. *mtdA* and *fch*; *fhs*, *fchA* and *folD*). qRT‐PCR analysis showed that these genes could not be transcribed at an identical level (Figure 3). The transcriptional levels of the MTHFD‐encoding genes (*folD* and *mtdA*) in the two engineered strains BL21‐WL and BL21(*ΔmetH*)‐C1T were lower than that of the FTHFL‐encoding genes (*fhs* and *ftfL*) and the FTHFC‐encoding genes (*fchA* and *fch*). Therefore, the MTHFD‐encoding gene may be an important factor limiting the increase in *L*‐5‐MTHF production. In addition, the analysis of metabolite accumulation showed that the amount of methenyl‐THF was much higher than that of THF and *L*‐5‐MTHF (Table 2), indicating that DHFR, FTHFL and FTHFC should not be the rate‐limiting enzymes in the constructed *L*‐5‐MTHF synthesis pathway (Figure 1). In the pathway, methenyl‐THF needs to be reduced by MTHFD to form methylene‐THF, which is further reduced by MTHFR to form *L*‐5‐MTHF. Therefore, the reason for the large accumulation of methenyl‐THF can be directed to the two steps. The measurement of the specific activity of MTHFD and MTHFR showed that both enzymes played a certain role in the engineered strains (Table 3). However, their activities were lower than the reported specific activity of MTHFD (2.4 U mg^−1^) in the cell extracts of *C. autoethanogenum* grown on fructose (Mock et al., 2015) and the specific activity of MTHFR (0.48 U mg^−1^) in the cell extracts of *E. coli* overexpressing MTHFR (Sheppard et al., 1999). It can be concluded that the low expression/activity of MTHFD is the key rate‐limiting factor in the constructed *L*‐5‐MTHF synthesis pathway. Although the *metH* gene in *E. coli* was knocked out, methionine could also be produced under the action of 5‐methyltetrahydropteroyltriglutamate‐homocysteine methyltransferase, which is encoded by the *metE* gene (Gonzalez et al., 1996). Therefore, Met and *S*‐AdoMet in cells still have inhibitory effects on MTHFR, which may also lead to low specific activity of MTHFR. Thus, other metabolic engineering strategies, including inserting strong promoters and ribosome binding site (RBS) sequences in the tandem genes encoding the rate‐limiting enzymes, screening the enzymes with high activities and without feedback inhibition, overexpressing glucose‐6‐phosphate dehydrogenase to strengthen the supply of NADPH, and introducing a reductive glycine pathway, could be considered in the future to improve the yield of *L*‐5‐MTHF (Bang & Lee, 2018; Kim et al., 2020; Shi et al., 2020).

In conclusion, by overexpressing endogenous enzymes (MTHFR and DHFR) of *E. coli*, the disruption of the gene encoding MTRR, which is involved in the consumption and synthesis inhibition of the target product, and the introduction of exogenous enzymes (FTHFL, FTHFC and MTHFD) from the one‐carbon metabolic pathway, greatly increased the production of *L*‐5‐MTHF in the engineered *E. coli* strains. The highest yield of 527.84 μg g^−1^ (1.24 mg L^−1^) was obtained for the engineered strain of BL21(*ΔmetH*)‐C1T after preliminary optimization, which was about 11.8 folds of that of the original strain. Although the yield remained lower than the reported maximum titre of *L*‐5‐MTHF in *Bacillus subtilis* (3.41 mg L^−1^) (Yang et al., 2022), the production reached a relatively high level in the engineered strains when using *E. coli* as the host. But the current level is not high enough for large‐scale industrial production, the metabolic engineering strategies such as fine‐tuning the enzymes associated with the C1 transfer pathway from *M. extorquens* AM1 or the Wood–Ljungdahl pathway from *C. autoethanogenum* are needed to further improve the production of *L*‐5‐MTHF in *E. coli*. This study paves the way for further metabolic engineering to achieve efficient biosynthesis of *L*‐5‐MTHF in *E. coli*.

## FUNDING INFORMATION

This work was supported by the National Natural Science Foundation of China (Grant Nos. 32170036 and 31470167).

## CONFLICT OF INTEREST

The authors declare no conflict of interest.

## Supporting information


Table S1
