# Supplementary material for: Biosynthesis of L‐5‐methyltetrahydrofolate by genetically engineered Escherichia coli
Source: Microb Biotechnol. 2022 Sep 7;15(11):2758–72. doi: 10.1111/1751-7915.14139 (PMC9618320; doi:10.1111/1751-7915.14139)
Supplement: Supplementary file 1 — Table S1 [file MBT2-15-2758-s001.docx]

# Supporting information

# Biosynthesis of *L*-5-methyltetrahydrofolate by genetically engineered *Escherichia coli*

Yubo Wang,^1^ Meng Zhang,^1^ Lexin Li,^1^ Jihong Yi,^1^ Jiyu Liang,^1^ Shuning Wang,^1*^ Ping Xu^2*^

^1^State Key Laboratory of Microbial Technology, Microbial Technology Institute, Shandong University, Qingdao 266237, People’s Republic of China

^2^State Key Laboratory of Microbial Metabolism, School of Life Sciences and Biotechnology, Shanghai Jiao Tong University, Shanghai 200240, People’s Republic of China

Running head: Engineering of *E. coli* for producing *L*-5-MTHF

* Correspondence:

Shuning Wang, [shuningwang@sdu.edu.cn](mailto:shuningwang@sdu.edu.cn), or Ping Xu, [pingxu@sjtu.edu.cn](mailto:pingxu@sjtu.edu.cn).

**Table S1 Primers used in this study**

| **Primer** | **Sequence (5'→3')^a^** |
| --- | --- |
| **Primers for vector construction** |  |
| *metF*_F | CATGCCATGGGCATGAGCTTTTTTCACG (NcoI) |
| *metF*_R | CCCAAGCTTTTATAAACCAGGTCGAACCC (HindIII) |
| *folA_*F | GGAATTCCATATGATCAGTCTGATTGCGGC (NdeI) |
| *folA*_R | CCGCTCGAGTTACCGCCGCTC (XhoI) |
| *ftfL*_F | CGCGGATCCATGCCCTCAGATATCGAGATC (BamHI) |
| *ftfL*_R | CCCAAGCTTCTAGAACAGCCCGTCGATC (HindIII) |
| *mf*_F | CGCCATATGTCCAAGAAGCTGCTCTTCCAG (NdeI) |
| *mf*_R | CGGGGTACCTCAGTTTACCTTGGACTTCACCGTC (KpnI) |
| CA1616.1618_F | GGAATTCCATATGACTTATAAATCAGAC (NdeI) |
| CA1616.1618_R | CCCTCGAGTTATAGGTTATTTTGTAAC (XhoI) |
| **Primers for *metH* gene knockout** |  |
| *metH*_PUF | TGGAATTGGGTAACCCGGCTTGTTGCGC |
| *metH*_PUR | aagcagctccagcctacacTTGTTCCACTTTGCTGCTCAC |
| *metH*_PKF | GTGAGCAGCAAAGTGGAACAAgtgtaggctggagctgctt |
| *metH*_PKR | GTCATACCCCAGATTCGGTGCatgggaattagccatggtcc |
| *metH*_PDF | ggaccatggctaattcccatGCACCGAATCTGGGGTATGAC |
| *metH*_PDR | TGCTCTGTACCAGTGCGGTAACGCCG |
| **Primers for qRT-PCR reaction** |  |
| q1618_F | CTATGGGACCTGTATTTG |
| q1618_R | TTGTTAGCAGCAGTGAGT |
| q1617_F | GCTATTATTGGAGCACAG |
| q1617_R | CAACTAAAGGTTCCGTCT |
| q1616_F | AAGGGTATTACTCCAAAG |
| q1616_R | TAACGACTGCCTCTATTC |
| q*ftfL*_F | TGTTCTGCCTCGCCAAGAAT |
| q*ftfL*_R | TTGAGGAGAACGGTCATCGC |
| q*mtdA*_F | GCGGCAAGGAGAAGCAGTCG |
| q*mtdA*_R | ATGCAGGACACGCGGAACG |
| q*fch*_F | TAACCTCACCATCGGCAAGAA |
| q*fch*_R | CATCACCGCGTCGAAAGC |
| q*16S*_F | TTCGGGAACCGTGAGACA |
| q*16S*_R | CTGGCAACAAAGGATAAGG |

^a^ Restriction enzyme sites are underlined, restriction endonucleases are shown in parentheses, and the lowercase letters are the homologous sequence of the target gene.


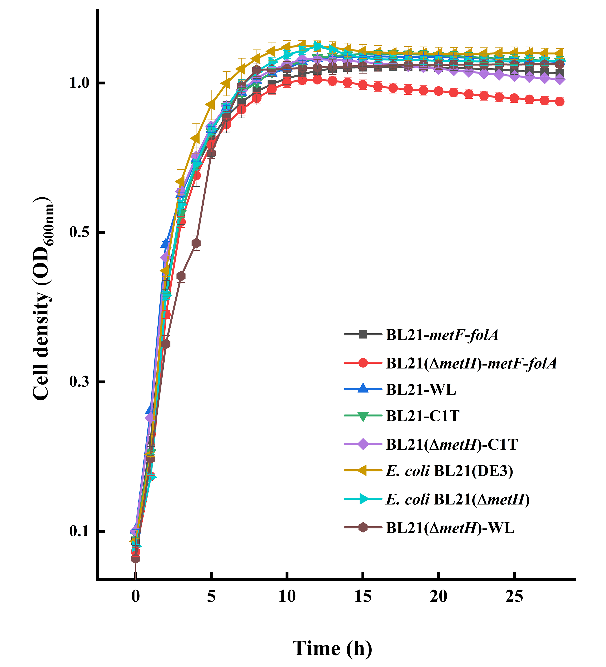


**Fig. S1.** Growth of the various engineered strains in TB medium without addition of any exogenous substances. The strains were cultivated in a microplate, and their growth was determined using a microplate reader (BioTek Instruments, USA). All data were the means of triplicate experiments, and the error bars indicate the standard deviations of the data.


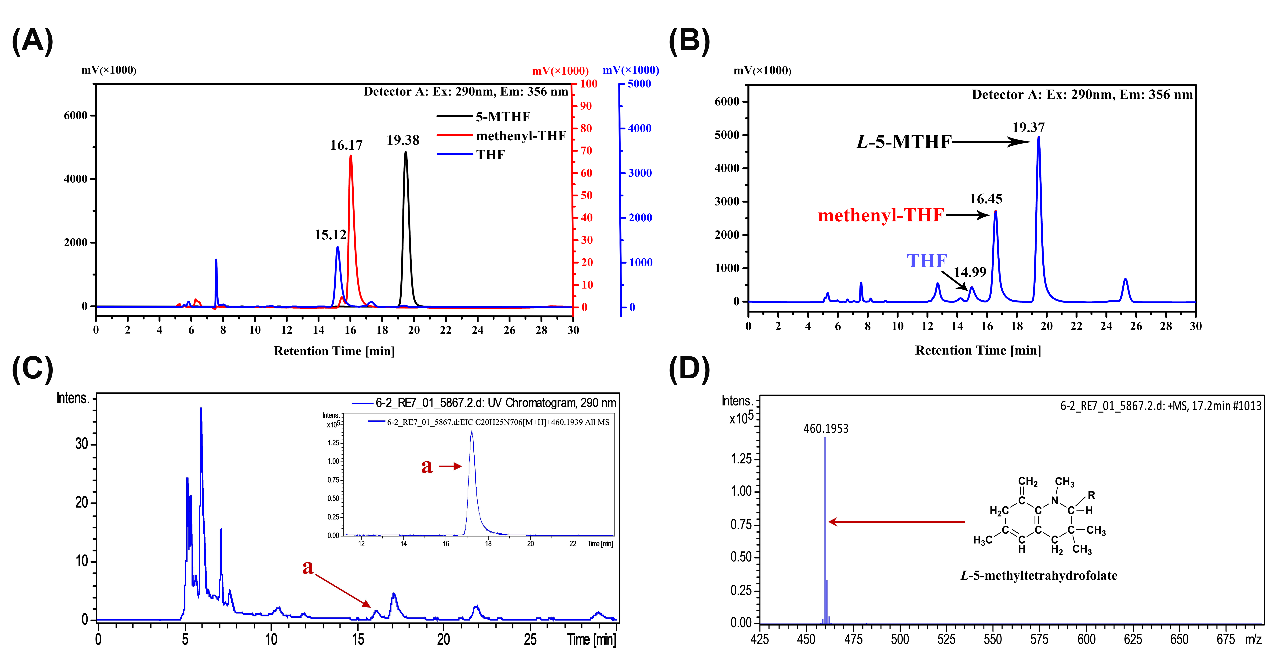


**Fig. S2.** HPLC and LC-MS profiles used for detecting *L*-5-MTHF in an *E. coli* BL21-WL cell extract. (A) Chromatograms of standards for 5-MTHF (black line), methenyl-THF (red line), and THF (blue line). (B) Chromatograms of the *E. coli* BL21-WL cell extract. (C) LC profile of the *E. coli* BL21-WL cell extract, where an enlargement of peak a is shown in the inset. A UV detector was used. (D) Mass spectrum of *L*-5-methyltetrahydrofolate (*m*/*z* 460.1953). In (A) and (B), the HPLC analysis used potassium phosphate-acetonitrile (93:7) as a mobile phase and a fluorescence detector (290/356 nm). In (C) and (D), the mobile phase was Milli-Q filtered water containing 8 mM formic acid and acetonitrile (93:7), and a UV detector (290 nm) was used for LC analysis. 5-MTHF: 5-methyltetrahydrofolate; methenyl-THF: methenyl-tetrahydrofolate; THF: tetrahydrofolate.


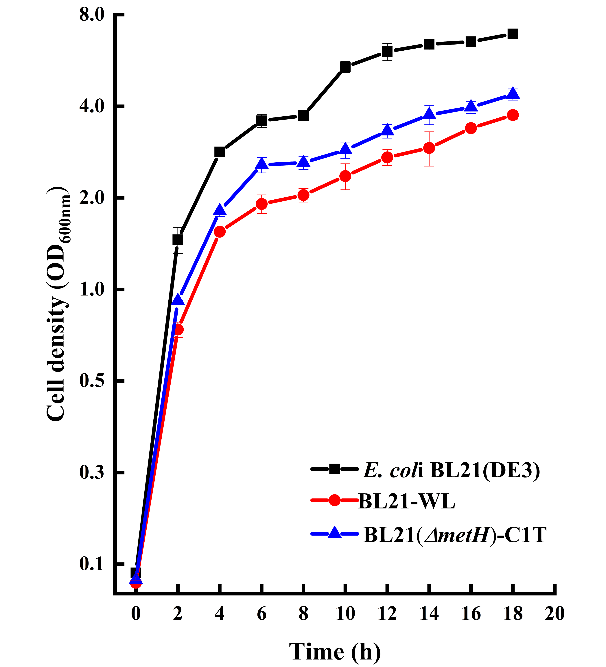


**Fig. S3.** Growth of *E. coli* BL21(DE3), Bl21-WL, and Bl21(*ΔmetH*)-C1T in TB medium containing folic acid, sodium formate, and IPTG. The strains were cultivated in 300-mL flasks, and their growth was determined using a photometer. All data were the means of triplicate experiments, and the error bars indicate the standard deviations of the data.
